# Scintillation Response Enhancement in Nanocrystalline Lead Halide Perovskite Thin Films on Scintillating Wafers

**DOI:** 10.3390/nano12010014

**Published:** 2021-12-21

**Authors:** Kateřina Děcká, Jan Král, František Hájek, Petr Průša, Vladimir Babin, Eva Mihóková, Václav Čuba

**Affiliations:** 1 Department of Nuclear Chemistry, Faculty of Nuclear Sciences and Physical Engineering, Czech Technical University in Prague, Břehová 7, 115 19 Prague, Czech Republic; kralja13@fjfi.cvut.cz (J.K.); vaclav.cuba@fjfi.cvut.cz (V.Č.); 2 Institute of Physics of the Czech Academy of Sciences, Cukrovarnická 10, 162 00 Prague, Czech Republic; hajek@fzu.cz (F.H.); petr.prusa@fjfi.cvut.cz (P.P.); babinv@fzu.cz (V.B.); mihokova@fzu.cz (E.M.); 3 Department of Solid State Engineering, Faculty of Nuclear Sciences and Physical Engineering, Czech Technical University in Prague, Břehová 7, 115 19 Prague, Czech Republic; 4 Department of Dosimetry and Application of Ionizing Radiation, Faculty of Nuclear Sciences and Physical Engineering, Czech Technical University in Prague, Břehová 7, 115 19 Prague, Czech Republic

**Keywords:** lead halide perovskites, nanocrystals, thin films, heterostructure, scintillator, fast timing, TOF-PET

## Abstract

Lead halide perovskite nanocrystals of the formula CsPbBr_3_ have recently been identified as potential time taggers in scintillating heterostructures for time-of-flight positron emission tomography (TOF-PET) imaging thanks to their ultrafast decay kinetics. This study investigates the potential of this material experimentally. We fabricated CsPbBr_3_ thin films on scintillating GGAG:Ce (Gd_2.985_Ce_0.015_Ga_2.7_Al_2.3_O_12_) wafer as a model structure for the future sampling detector geometry. We focused this study on the radioluminescence (RL) response of this composite material. We compare the results of two spin-coating methods, namely the static and the dynamic process, for the thin film preparation. We demonstrated enhanced RL intensity of both CsPbBr_3_ and GGAG:Ce scintillating constituents of a composite material. This synergic effect arises in both the RL spectra and decays, including decays in the short time window (50 ns). Consequently, this study confirms the applicability of CsPbBr_3_ nanocrystals as efficient time taggers for ultrafast timing applications, such as TOF-PET.

## 1. Introduction

Lead halide perovskite nanocrystals of the formula CsPbX_3_ (X = Cl, Br, I) were first reported more than 20 years ago [1,2,3], but have only been studied thoroughly since 2015, when their colloidal synthesis was introduced [4]. There are a large body of papers published on this topic, but the majority of work focuses on their luminescent properties and applications such as LEDs, displays, photovoltaics, or lasers [5,6,7].

However, their properties such as fast decay times, narrow emission bands, and high light yield are also desirable for scintillation detectors. Some papers have been published on this topic [8,9,10,11,12,13,14], but not nearly as many.

Moreover, in contrast with, e.g., CsPbBr_3_ single crystals [11], CsPbBr_3_ nanocrystals show negative thermal quenching (increase of radioluminescence intensity with increasing temperature) leading to scintillation light yield of 24,000 ± 2100 MeV^−1^ at 300 K under 662 keV excitation, which is one order of magnitude higher than other nanocrystals in this family, namely FAPbBr_3_ and CsPbI_3_ [15].

The application of any nanocrystals as prospective scintillators has some common issues, among which the most serious is their poor stopping power. Simple calculation shows that the half-value layer of CsPbBr_3_ for Cu K_α_ line is ~7.5 μm and for Bremsstrahlung generated in X-ray tube operating at 40 kV the half-value layer is ~100 μm (see Supplementary Information for details and used values). This means that nanocrystals must be in the form of a sufficiently thick film to stop at least some of the incident radiation. Moreover, these values represent only the lower limit of a rough estimate, because they do not take into consideration the reduction of density due to the presence of surface ligands and the lower density of nanomaterials compared to their bulk counterparts. Therefore, real half-values will be even larger.

To fabricate high quality thin films of such thicknesses is not an easy task by itself. Moreover, in such thick films one can expect serious issues with self-absorption, because semiconductor nanocrystals have generally small Stokes shifts and CsPbX_3_ nanocrystals are no exception. Small Stokes shift can be overcome by introducing a wavelength shifter to the mixture [16], but it will inevitably lead to longer rise and decay times, which is undesirable for some applications requiring fast response, such as time-of-flight positron emission tomography (TOF-PET) or high energy physics.

It has been proposed and explored before, that a sandwich-like structure combining the bulk scintillator with high stopping power and semiconductor nanocrystals with ultrafast decay times is highly promising for TOF-PET detectors [17,18]. The bulk scintillator serves as a stopping medium and provides the energy resolution and nanocrystals serve as time taggers.

In this work we fabricate similar, but much simpler composite materials; CsPbBr_3_ thin films on GGAG:Ce (Gd_3_(Al,Ga)_5_O_12_:Ce) scintillating wafer. GGAG:Ce is a modern scintillator that possesses high stopping power (effective atomic number Z_eff_ = 54) and high light yield, slightly under 60,000 MeV^−1^ when optimized [19,20]. We use lower energy X-rays to characterize this nanocomposite as the first step towards future study of CsPbBr_3_ on GGAG:Ce sandwich pixel under 511 keV gamma-rays excitation. We show an enhancement effect between these two materials that leads to improved radioluminescence intensities (higher than a simple sum of individual emissions), while preserving the sub-nanosecond decay components of CsPbBr_3_ nanocrystals both in photo- and radioluminescence decays.

## 2. Materials and Methods

### 2.1. Chemicals

Cs_2_CO_3_ (99.9%, Sigma-Aldrich, Saint Louis, MO, USA), PbBr_2_ (99.999%, Sigma-Aldrich), oleylamine (OAm, 70%, Sigma-Aldrich), oleic acid (OA, 90%, Sigma-Aldrich), 1-octadecene (90%, Sigma-Aldrich), toluene (99.8%, Sigma-Aldrich), didodecyldimethylammonium bromide (DDAB, 98%, Sigma-Aldrich), and ethylacetate (p. a., PENTA, Prague, Czech Republic). All chemicals were used as received without further purification, unless stated otherwise.

### 2.2. Wafers for Thin Films

We used two types of wafers for thin films deposition: a commercially available glass slide as a non-scintillating wafer (square, 18 mm × 18 mm × 0.17 mm, Hirschmann, Eberstadt, Germany) and GGAG:Ce as a scintillating wafer (circle, ~15 mm in diameter and 0.2 mm thick). The GGAG:Ce (Gd_2.985_Ce_0.015_Ga_2.7_Al_2.3_O_12_) was grown at the Czech Academy of Sciences.

### 2.3. CsPbBr_3_ Synthesis

To synthetize CsPbBr_3_ nanocrystals, the standard hot-injection (HI) procedure introduced by Protesescu et al. was used [4]. The preparation of cesium oleate was modified to increase Cs:OA ratio in the reaction to 1:5 according to the study by Lu et al. [21]. In short, 0.752 mmoL of PbBr_2_, 20 mL of 1-octadecene (ODE), 2 mL of oleylamine (OAm), and 1.78 mL of oleic acid (OA), were mixed in 100 mL 3-neck flask and degassed at 110 °C under vacuum for 1 h. After that, 0.5 mL of dried pre-synthesized cesium oleate solution (0.4 M) was injected at 170 °C under argon atmosphere. More details on the CsPbBr_3_ synthesis can be found in our previous publication [22].

Ligand exchange reaction was performed following the procedure presented by Imran et al. [23], and all exchange reactions were performed at room temperature in air. The crude reaction mixture from HI synthesis was mixed with 55 mM DDAB toluene solution (volume ratio 3:2) and vigorously stirred for 2 min. Thereafter, NCs were precipitated by addition of ethyl acetate (15 mL per 3 mL of crude reaction mixture) and isolated by centrifugation for 10 min at 4800× *g*. Final CsPbBr_3_ solution was obtained by redispersion in toluene.

For preparation of CsPbBr_3_ thin films, the solution concentration was adjusted to 45−50 mg⋅mL^−1^. The NC concentration was determined from the solution absorbance at 400 nm according to Maes et al. [24].

### 2.4. Thin Film Fabrication

CsPbBr_3_ thin films were fabricated using the spin-coating technique, two different processes of repeated spin-coating were developed to prepare thicker films.

In the static process the solution was repeatedly deposited on stationary wafer followed by rotation at 2000 rpm for 1 min. Films on the glass wafer were prepared by depositing 40 µL of solution 40× (to compare with the dynamic process) or 50× (for the rest of experiments), the film on scintillating wafer was fabricated by deposition of 20 µL repeated 50×. Smaller amount of solution (20 μL) was used because GGAG:Ce wafer is smaller than the glass wafer.

In the dynamic process the solution was deposited dropwise on constantly rotating substrate. Spacing between individual drops was 45 s, rotation rate was 2000 rpm. To fabricate the CsPbBr_3_ film on the glass slide, 600 µL of the solution was used; the thin film on scintillating wafer was prepared using 500 µL of the solution.

### 2.5. Characterization

X-ray powder diffraction (XRPD) was measured using a Rigaku Miniflex 600 diffractometer equipped with the Cu X-ray tube (average wavelength K_α1,2_ 0.15418 nm, voltage 40 kV, current 15 mA). Data were collected with a speed of 2°/min and compared with the ICDD PDF-2 database, version 2013. The Halder–Wagner method with Scherrer constant value 0.94 was used for the determination of the linear crystallite size. The scanning electron microscopy (SEM) was obtained using an FEI XL30 ESEM microscope with home-build cathodoluminescence setup for measurement spectrally and spatially resolved cathodoluminescence. It consists of optical system for light collection, single-grating monochromator, and photomultiplier tube Hamamatsu H7711-13. Width of slits enable better than 20 nm spectral resolution. Absorption spectra were collected using a Cary 100 spectrophotometer (Varian, Palo Alto, CA, USA). Photoluminescence (PL) excitation and emission spectra were collected using a FluoroMax spectrofluorometer (Horiba Jobin Yvon, Kyoto, Japan). Radioluminescence (RL) spectra were collected using a 5000M spectrofluorometer (Horiba Jobin Yvon) with a monochromator and TBX-04 (IBH, Glasgow, Scotland) photodetector, the excitation source was a Seifert X-ray tube (40 kV, 15 mA). RL decay curves were collected using the hybrid picosecond photon detector HPPD-860 and Fluorohub unit (Horiba Scientific, Kyoto, Japan). Samples were excited by the picosecond (ps) X-ray tube N5084 from Hamamatsu, operating at 40 kV. The X-ray tube was driven by the ps light pulser (Hamamatsu, Hamamatsu City, Japan) equipped with a laser diode operating at 405 nm. The instrumental response function FWHM of the setup is about 76 ps. Convolution procedure was applied to all decay curves to determine true decay times (SpectraSolve 3.01 PRO software package, Ames Photonics, Fort Worth, TX, USA). The contribution of a component expressed as a percentage (often referred to as a light sum, LS) was calculated as:

$${\mathrm{LS}}_{n}=\frac{A_{n}\tau_{n}}{\sum A_{i}\tau_{i}}$$

where *A_n_* and *τ_n_* denote the amplitude and decay time of the nth component. All luminescence measurements were performed using similar configuration, the emission was detected from the same surface where excited.

## 3. Results and Discussion

**Figure 1:**
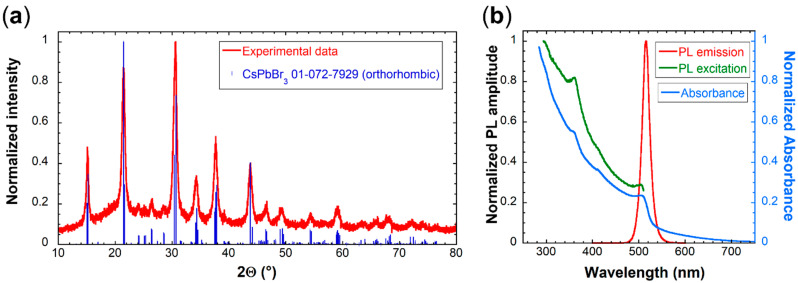
(**a**) XRPD pattern (red line) of synthesized material used for spin-coating. Identified phase according to ICDD PDF-2 database was orthorhombic CsPbBr_3_ no. 01-072-7929 (blue lines). (**b**) PL emission (red line), excitation (green line), and absorption (blue line) spectra of synthesized solution used for spin-coating.

CsPbBr_3_ samples used for the thin film fabrication were analyzed using the X-ray powder diffraction (XRPD), photoluminescence emission (PL), and excitation (PLE) spectra, as well as absorption spectra (see Figure 1).

XRPD analysis in Figure 1 shows that synthesized nanocrystals were pure orthorhombic CsPbBr_3_ phase with the mean crystallite size of (13.8 ± 0.6) nm, which is consistent with the value obtained by XRPD and TEM analysis in our previous paper [22]. The phase purity is further confirmed by the PLE spectrum in Figure 1b lacking the dip at 310 nm, and absorption spectrum lacking the peak at the same wavelength, which are both characteristic of the Cs_4_PbBr_6_ impurity [22,25,26]. The PL spectrum shows a single excitonic peak at 515 nm. More detailed characterization of the same type of samples prepared before can be found in our recently published work [22].

**Figure 2:**
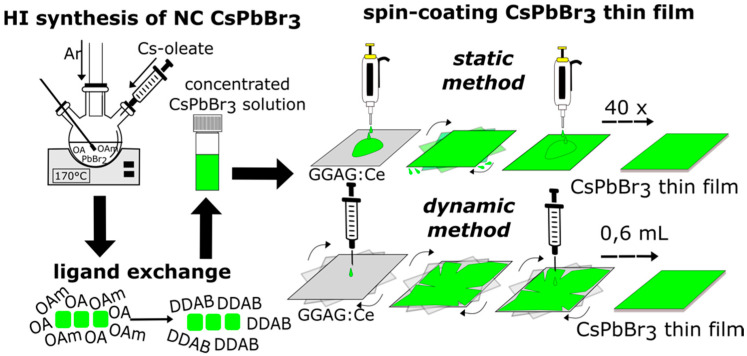
Schematic representation of the synthesis and spin-coating processes; hot injection (HI) synthesis, ligand exchange of oleic acid (OA) and oleylamine (OAm) for dioleyldimethylammonium bromide (DDAB) and static and dynamic spin-coating processes.

CsPbBr_3_ for the thin film fabrication had to be surface modified using a ligand exchange procedure of oleic acid and oleylamine for dioleyldimethylammonium bromide (DDAB) [23]. Without the ligand exchange, thicker films with higher radioluminescence (RL) intensity could not be prepared, see Figure S1 in Supplementary Information. DDAB capping allowed repeated spin-coating process in order to increase the film thickness, see Figures S2–S4 in Supplementary Information. Figure 2 shows two optimized processes for fabrication of thin films with sufficient thickness.

**Figure 3:**
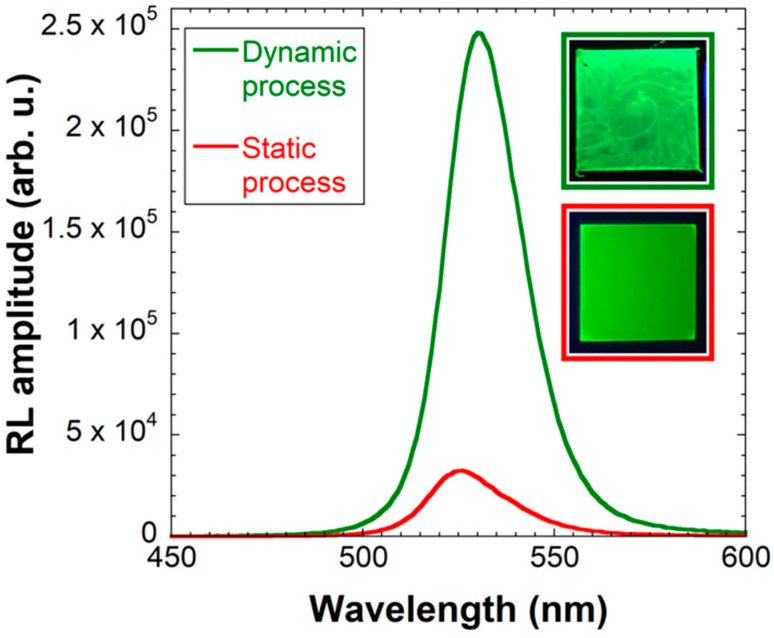
RL spectra of CsPbBr_3_ thin films on glass prepared by using 0.6 mL of CsPbBr_3_ solution in the dynamic process (green line) and by stacking 40 layers in the static process (red line). Inset: Photographs under UV illumination of the film prepared by the dynamic process (top) and static process (bottom).

Figure 3 displays RL spectra of CsPbBr_3_ films prepared by both methods. It is clear that the dynamic process yields the film with higher RL intensity. Even better, this film was prepared with significantly lower amount of material (0.6 mL for the dynamic process vs. 1.6 mL for 40 layers of the static process), which significantly reduces its cost. On the other hand, the static process yields the film of much higher homogeneity even to the naked eye as evidenced by the photographs in the inset of Figure 3.

Please note that the RL spectra of thin films are red-shifted compared to the PL spectrum of CsPbBr_3_ nanocrystals in Figure 1b. This shift is probably caused by different excitation process under X-rays, and also by reabsorption inevitably occurring in the CsPbBr_3_ layer due to its small Stokes shift, as discussed in the Introduction section.

A question to be answered is whether a good homogeneity of the thin film is that important for the intended application in TOF-PET, where crucial requirements are the high light output and fast response (i.e., fast rise and decay times) [18]. To find the answer, both processes were used to prepare films on scintillating GGAG:Ce wafer and both RL spectra and decays were measured.

The mean measured thickness of the film prepared by the static process was ~3 μm (5 spots, 2.4 μm–3.7 μm) and for the dynamic process it was also ~3 μm, but with much wider distribution (12 spots, 1.08 μm–5.58 μm) (see Figures S4 and S5 in Supplementary Information for relevant SEM images).

**Figure 4:**
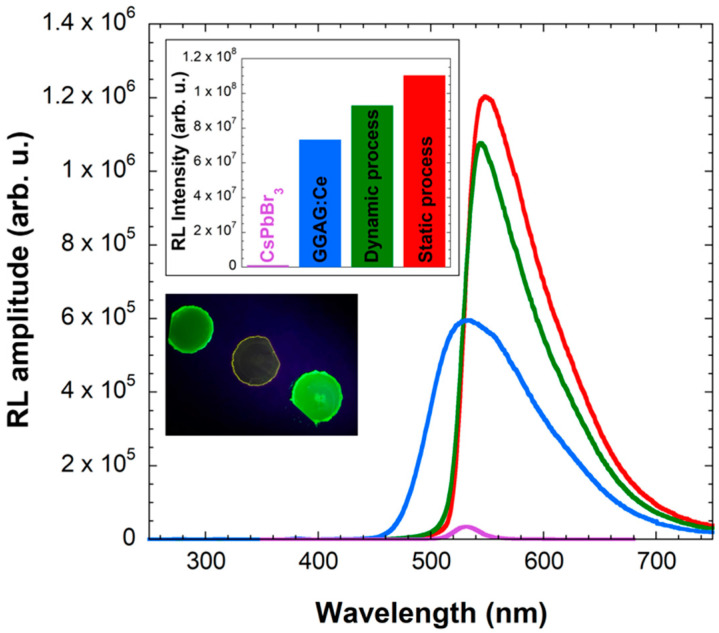
RL spectra of prepared CsPbBr_3_ films on glass by the static process (purple line) and on GGAG:Ce by the static process (red line) and the dynamic process (green line), compared to the pure GGAG:Ce wafer (blue line). Graph in the inset: Integrated RL intensities of presented spectra. Photograph in the inset, from left to right: CsPbBr_3_ film on GGAG:Ce prepared by the static process, pure GGAG:Ce wafer, CsPbBr_3_ film on GGAG:Ce prepared by the dynamic process. Note that UV illumination intensity is not homogeneous among the samples.

Figure 4 shows RL spectra of CsPbBr_3_ films prepared by both processes compared to a pure GGAG:Ce plate and pure CsPbBr_3_ film on glass prepared by the static process. CsPbBr_3_ films on GGAG:Ce prepared by both methods show significantly larger RL intensity than both pure GGAG:Ce and pure CsPbBr_3_ on glass, even if part of the GGAG:Ce emission (below ~530 nm) is absorbed by the CsPbBr_3_ layer (cf. absorption spectrum in Figure 1). Please note that the quantitative comparison to the film on glass is not entirely appropriate because of the size difference in the wafers. The glass wafer is larger, therefore its luminescence intensity is actually overvalued, which further illustrates the relatively low RL intensity of the pure CsPbBr_3_ film on glass wafer.

The overall intensity of the nanocomposite is in both cases (the static and the dynamic processes) higher than a simple sum of the two individual emissions. The shape of the RL spectra indicate that both CsPbBr_3_ and GGAG:Ce emissions are enhanced. The CsPbBr_3_ emission is probably enhanced by the absorption and subsequent reemission of the GGAG:Ce emission. However, the enhancement of GGAG:Ce emission cannot be explained easily.

Interestingly, in contrast to previous results in Figure 3, there is not much difference in RL spectra of CsPbBr_3_ films prepared by different methods on GGAG:Ce wafer. At this point, it seems that the answer to our question is that the homogeneity of the fabricated film does not play a significant role in the overall RL intensity of the final nanocomposite.

**Figure 5:**
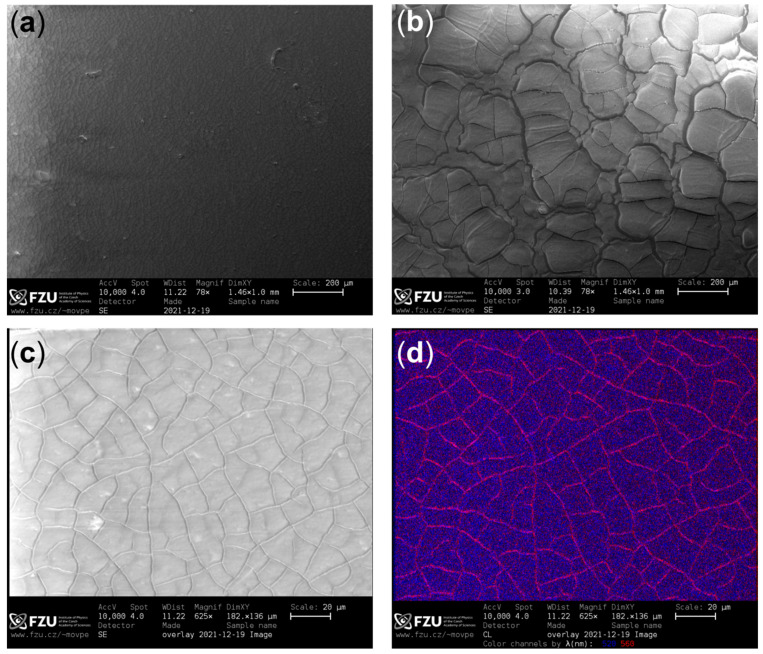
SEM images of the CsPbBr_3_ film on GGAG:Ce prepared by the static (**a**) and dynamic (**b**) methods at very low magnification (78×). SEM image (**c**) and cathodoluminescence (CL) image (**d**) of the film prepared by the static method at larger magnification (625×). Red color in the CL image represents the 560 nm light (which is emitted from GGAG:Ce) and blue color the 520 nm light (which is emitted mostly by CsPbBr_3_).

The enhancement of the GGAG:Ce emission can be explained by analyzing SEM pictures and cathodoluminescence data, see Figure 5. Micrographs at very low magnification (78×, Figure 5a,b) confirm that the thin film prepared using the dynamic process has poor homogeneity and very large cracks. SEM image in Figure 5c shows the static thin film at higher magnification (625×), which reveals that this film also has cracks, but much thinner. Figure 5d (cathodoluminescence image) shows that the 560 nm light, which is emitted solely by GGAG:Ce, is shining with high intensity through the cracks. This phenomenon can explain the enhancement of the wafer’s RL response in Figure 4; the cracks probably serve as a light guide for its emission. Similar effect has been observed before [27] and is even investigated as a way for deliberate increase of light extraction [28].

**Figure 6:**
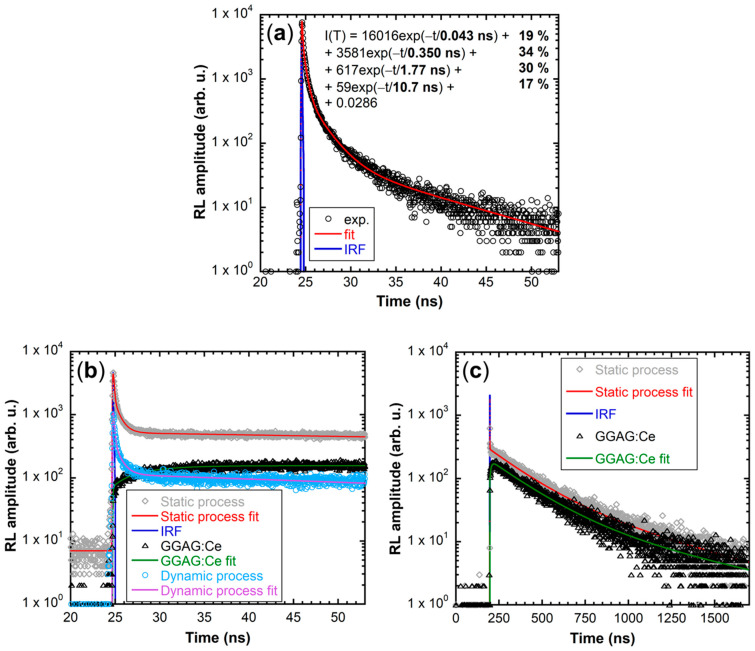
(**a**) Scintillation decay of the pure CsPbBr_3_ film prepared by the static process (50 layers) on glass. (**b**) Scintillation decays in the short time window of CsPbBr_3_ films on GGAG:Ce prepared by the static process (grey diamonds, red line) and the dynamic process (light blue circles, purple line) compared to the scintillation decay of pure GGAG:Ce wafer (black triangles, green line). (**c**) Scintillation decays in the long time window of the CsPbBr_3_ film on GGAG:Ce prepared by the static method (grey diamonds, red line) compared to the scintillation decay of the pure GGAG:Ce (black triangles, green line). Blue line represents the instrumental response function (IRF) in all graphs.

Figure 6 shows scintillation decay times of CsPbBr_3_ films prepared by both methods on GGAG:Ce wafers with comparison to the film prepared by the static method on glass and the pure GGAG:Ce wafer. All the decays were recorded in both short (50 ns, Figure 6a,b) and long (2 μs, Figure 6c) time windows. Decays in the short time window are of great importance for the target application in TOF-PET, because even the fastest sub-nanosecond decay components are well resolved.

Scintillation decay in the panel (a) in Figure 6 was measured for a qualitative comparison of the pure CsPbBr_3_ film on glass to films on GGAG:Ce. It demonstrates the applicability of CsPbBr_3_ films as ultrafast scintillators, because more than 50% of the scintillation light is emitted within the sub-nanosecond time gate. Scintillation decays in the panel (b) demonstrate that the ultrafast CsPbBr_3_ emission is preserved even if the film is fabricated on the scintillation wafer, as well as the slow emission on GGAG:Ce (this component is resolved only in the long time window in Figure 6c).

The comparison of static and dynamic processes confirms the trend already observable in RL spectra (Figure 4), namely, that the static process results in the film with higher overall RL intensity when combined with the GGAG:Ce scintillator. Similarly as in RL spectra in Figure 4, we also observed significant enhancement of GGAG:Ce emission on the sample prepared by the static process caused by the light-guiding effect on cracks, as discussed above and demonstrated in Figure 5. Interestingly, this enhancement is no longer observable in the sample prepared by the dynamic process. This phenomenon requires more thorough study in the future, but our preliminary conclusion and the answer to our question is that some level of film homogeneity, which ultimately was not achieved by the dynamic process, is probably needed for the light-guiding effect.

Figure 6c shows the scintillation decay of pure GGAG:Ce compared to the sample prepared by the static process. It displays the long GGAG:Ce decay component and further confirms the enhancement of the GGAG:Ce emission thanks to the CsPbBr_3_ film prepared by the static process. Decay of the sample prepared by the dynamic process is not presented because it completely overlaps with both presented decays but can be found in Figure S6 in the Supplementary Information along with the fit parameters of both CsPbBr_3_ films on GGAG:Ce.

Summary of the fit rise and decay times can be found in Table 1. Fit parameters of the pure CsPbBr_3_ film on glass can be found in Figure 6a.

## 4. Conclusions

We prepared CsPbBr_3_ films on both the glass and GGAG:Ce scintillating wafers with the target application in TOF-PET. We compared two methods for the film preparation, the static and the dynamic processes. While the dynamic process is more effective in terms of material waste, the static process yields much more homogeneous films. This was found important for the intended application because the sample on GGAG:Ce exhibited higher intensity in RL spectra and especially in scintillation decays.

Moreover, we demonstrated a synergic effect by combining CsPbBr_3_ nanoscintillator and GGAG:Ce bulk scintillator. The resulting composite exhibited enhanced RL intensity while preserving the ultrafast CsPbBr_3_ decay. Consequently, the thin nanocomposite layer is able to perform as an efficient time tagger in a sampling detector geometry. We can conclude, that presented material combination is indeed a potential candidate in the sandwich detector for ultrafast timing applications, such as TOF-PET.

## Figures and Tables

**Table 1:** Summary of fit rise times and decay times of pure GGAG:Ce measured in the long time window and CsPbBr_3_ films on GGAG:Ce prepared by both static and dynamic processes in the short time window. Long components of GGAG:Ce could not be resolved in the short time window.

Sample	Rise Time	Decay Time	Light Sum
GGAG:Ce	8 ns	200 ns	63%
		660 ns	37%
Static process	50 ps	80 ps	1%
		700 ps	1%
		long	98%
Dynamic process	30 ps	120 ps	3%
		770 ps	2%
		long	95%

## Data Availability

Data presented in this study are available on request from the corresponding author.

## Supplementary Materials

The following are available online at https://www.mdpi.com/article/10.3390/nano12010014/s1, Calculation of the half-value layers, Figure S1: RL spectra of CsPbBr_3_ thin films capped with oleic acid and oleylamine on the glass wafer with increasing number of depositions (1–40 layers), Figure S2: RL spectra of CsPbBr_3_ thin films capped with didodecyldimethylammonium bromide on the glass wafer with increasing number of depositions (1–40 layers), Figure S3: Linear dependence of the RL intensity in Figure S2 on the number of layers deposited by the static spin-coating process, Figure S4: SEM images of the CsPbBr_3_ thin film edge. Sample was prepared on the GGAG:Ce wafer, 50 layers deposited by the static process, Figure S5: SEM images of the CsPbBr_3_ thin film edge. Sample was prepared on the GGAG:Ce wafer, 0.6 mL deposited by the dynamic process, Figure S6: Scintillation decays in the long time window of CsPbBr_3_ thin films on GGAG:Ce prepared by the static process (left) and the dynamic process (right). Black circles represent experimental data, red line represents the fit and blue line is the instrumental response function (IRF). Reference [29] is cited in the Supplementary Material.
