# Supplementary material for: Scintillation Response Enhancement in Nanocrystalline Lead Halide Perovskite Thin Films on Scintillating Wafers
Source: Nanomaterials (Basel). 2021 Dec 21;12(1):14. doi: 10.3390/nano12010014 (PMC8746850; doi:10.3390/nano12010014)
Supplement: Supplementary file 1 [file nanomaterials-12-00014-s001.zip › nanomaterials-1493212-supplementary.pdf]

Supplementary information

Scintillation response enhancement in nanocrystalline lead halide perovskite thin films on scintillating wafers

Kateřina Děcká [1,2,*], Jan Král [1], František Hájek [2,3], Petr Průša [2,4], Vladimir Babin [2], Eva Mihóková [2,3] and Václav Čuba [1]

1 Department of Nuclear Chemistry, Faculty of Nuclear Sciences and Physical Engineering, Czech Technical University in Prague, Břehová 7, 115 19 Prague 1, Czech Republic;
2 Institute of Physics of the Czech Academy of Sciences, Cukrovarnická 10, 162 00 Prague 6, Czech Republic;
3 Department of Solid State Engineering, Faculty of Nuclear Sciences and Physical Engineering, Czech Technical University in Prague, Břehová 7, 115 19 Prague 1, Czech Republic;
4 Department of Dosimetry and Application of Ionizing Radiation, Faculty of Nuclear Sciences and Physical Engineering, Czech Technical University in Prague, Břehová 7, 115 19 Prague 1, Czech Republic;;
* Correspondence: Katerina.Decka@fjfi.cvut.cz;

Half value layers for $CsPbBr_3$

The thickness of $CsPbBr_3$ layer needed to reduce X-ray intensity to 50% of its initial value was calculated as:

$$x_{1/2} = \frac{\ln 2}{\rho \cdot \mu_m}$$

where ρ = 4.83 g·cm^{-3} is the density of orthorhombic $CsPbBr_3$ (PDF card 01-072-7929 from ICDD PDF-2 database) and the total mass attenuation coefficient without scattering μ_m was obtained from the NIST Standard Reference Database 8 [29]. For Cu K_α line (μ_m = 188.8 cm^2·g^{-1}) the half-value layer of $CsPbBr_3$ was determined to be 7.6 μm, calculated half-value layer for 40 keV Bremsstrahlung (μ_m = 13.3 cm^2·g^{-1}) was 107.9 μm.

RL spectra before and after ligand exchange

Figure S1 shows radioluminescence (RL) spectra of samples prepared by the static spin-coating process (1–40 repetitions), but without the ligand exchange step, i. e. $CsPbBr_3$ nanocrystals were capped with oleic acid and oleylamine. RL is weak and does not increase with increasing number of deposited layers.

Figure S2 displays the RL spectra of analogously prepared samples, only using $CsPbBr_3$ nanocrystals capped with dioleyldimethylammonium bromide (DDAB). The RL intensity (integrated RL spectrum) increases linearly with the number of deposited layers, see Figure S3.

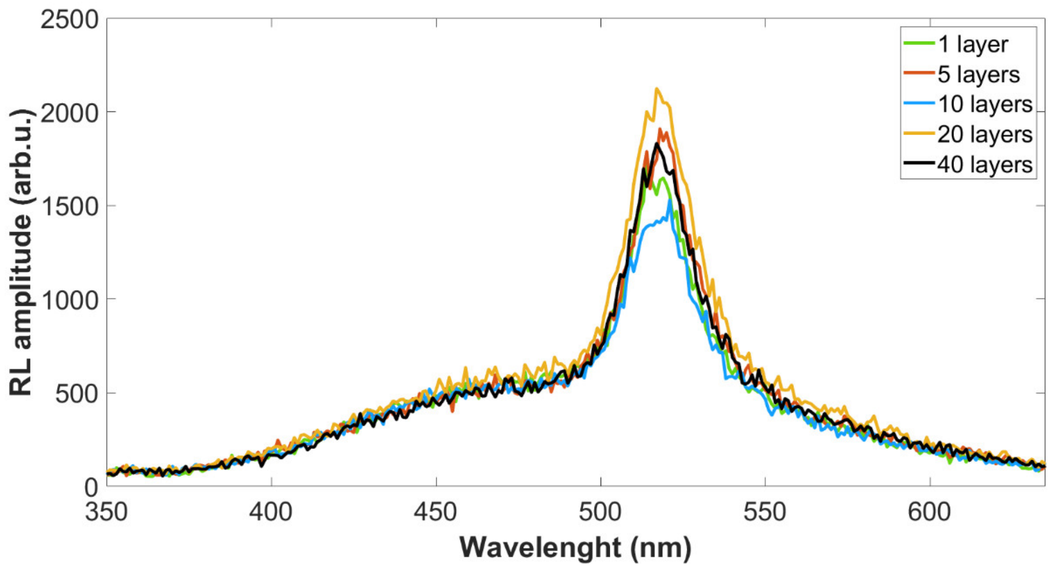

Figure S1. RL spectra of $CsPbBr_3$ thin films capped with oleic acid and oleylamine on the glass wafer with increasing number of depositions (1–40 layers).

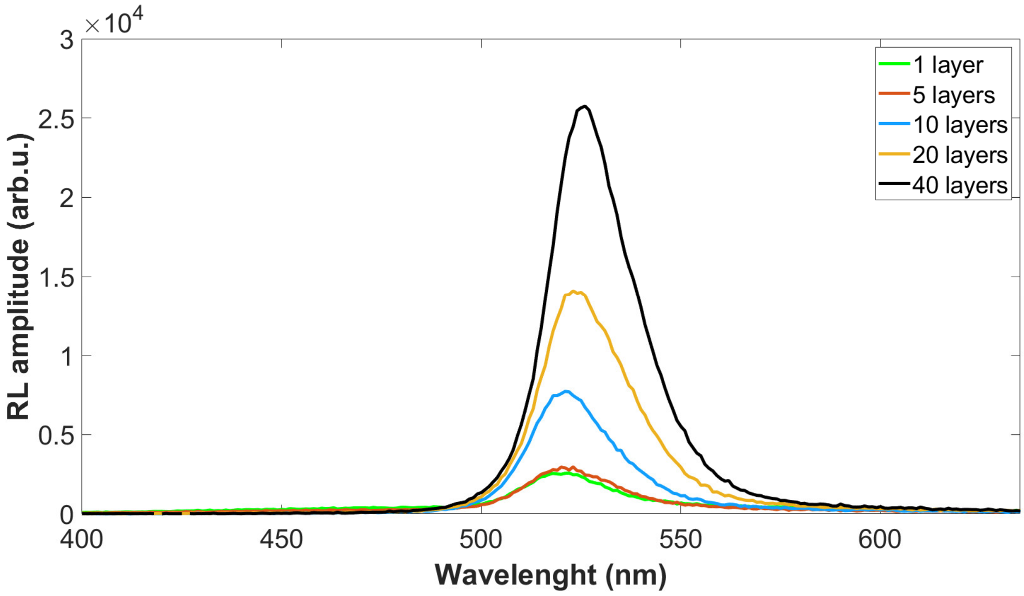

Figure S2. RL spectra of $CsPbBr_3$ thin films capped with didodecyldimethylammonium bromide on the glass wafer with increasing number of depositions (1–40 layers).

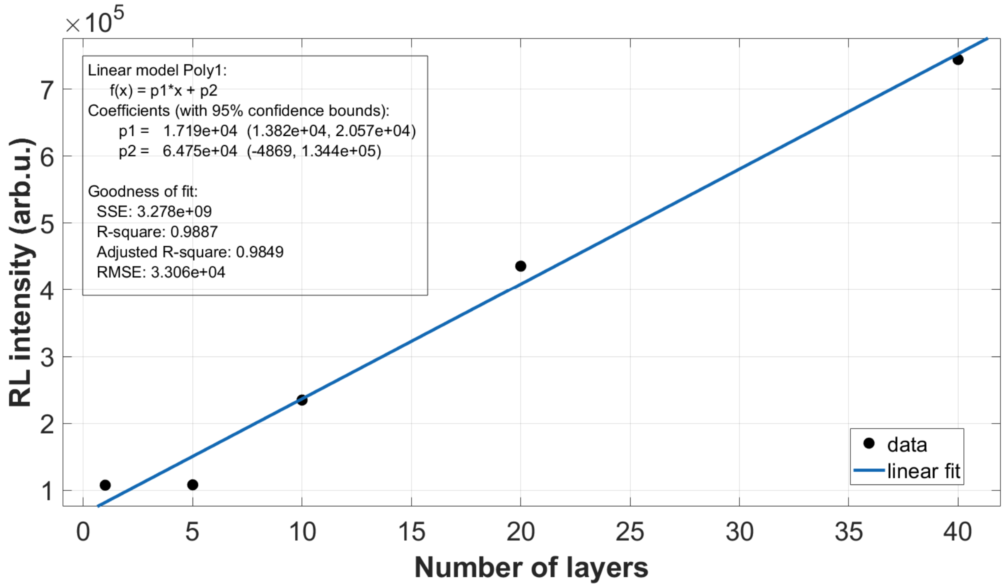

Figure S3. Linear dependence of the RL intensity in Figure S2 on the number of layers deposited by the static spin-coating process.

Film thicknesses

To determine the thin film thickness, samples were broken and the edge was analyzed using the SEM imaging. Figure S5 shows the edge of the thin film prepared by the static process (two different spots) and Figure S6 sample prepared by the dynamic process (three different spots). The homogeneity of the thin film prepared by the static process is much better.

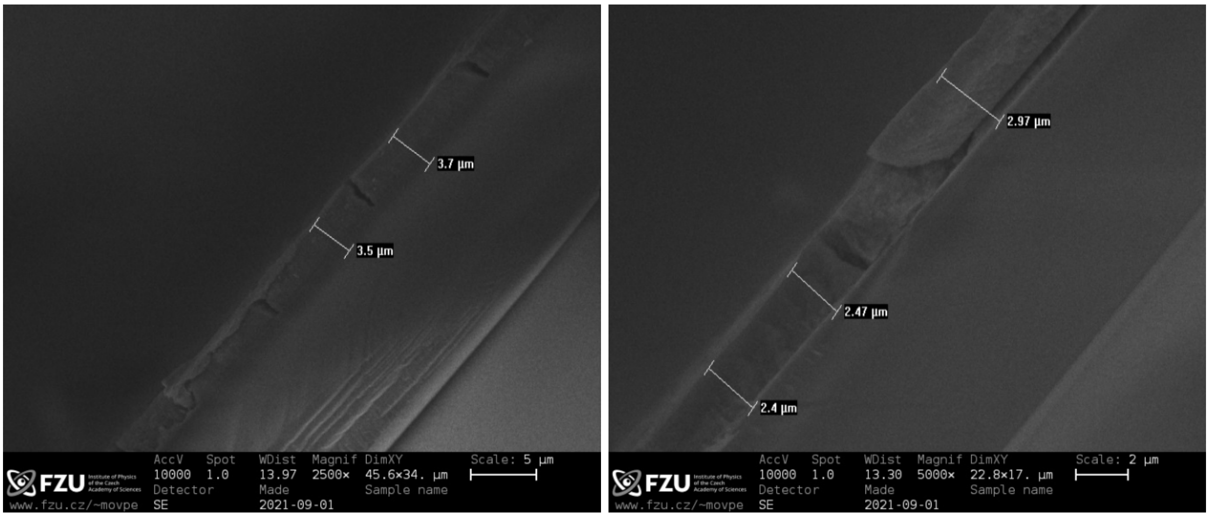

Figure S4. SEM images of the $CsPbBr_3$ thin film edge. Sample was prepared on the GGAG:Ce wafer, 50 layers deposited by the static process.

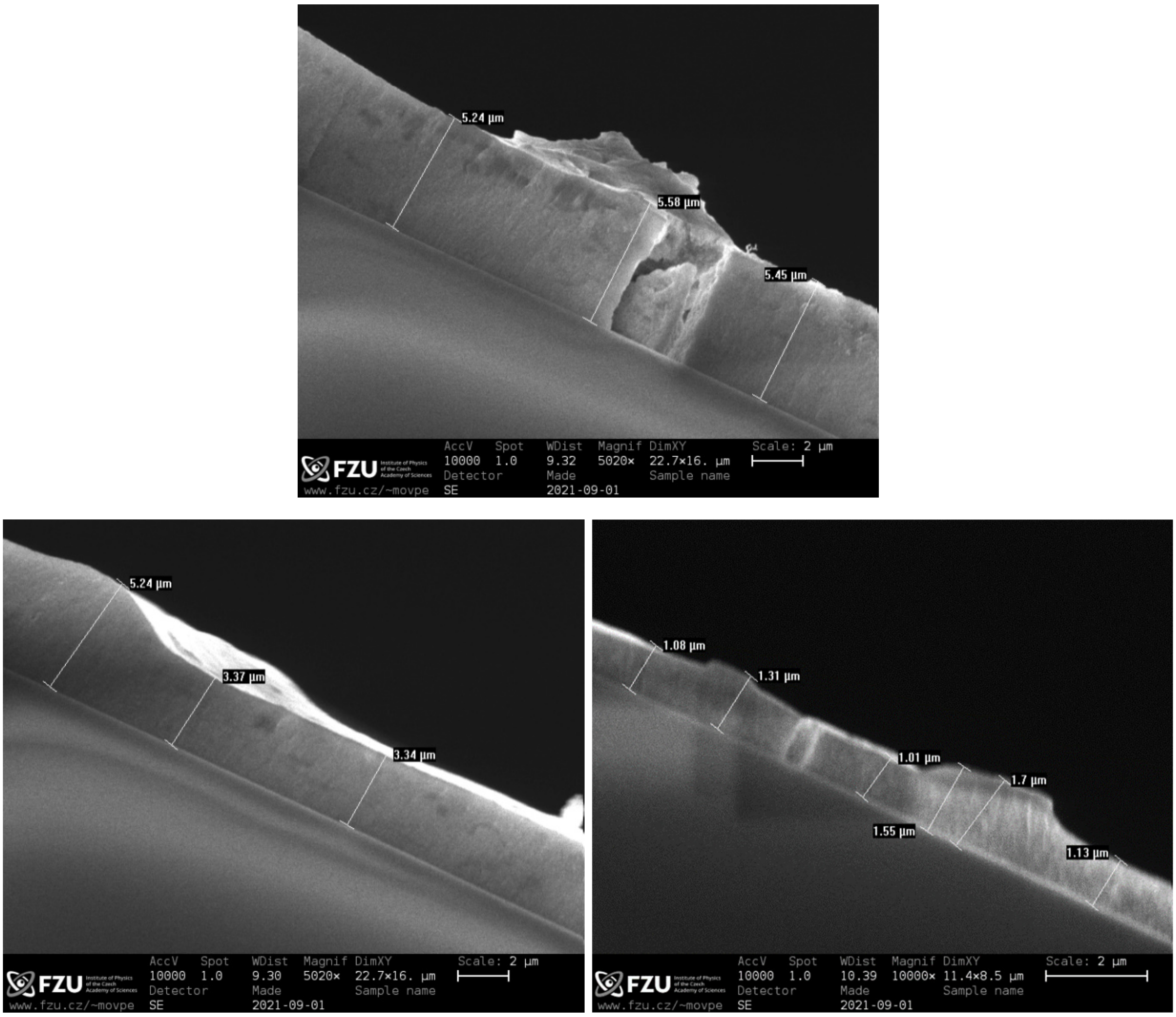

Figure S5. SEM images of the $CsPbBr_3$ thin film edge. Sample was prepared on the GGAG:Ce wafer, 0.6 ml deposited by the dynamic process.

Scintillation decays of samples prepared by the static and the dynamic processes in the long time window

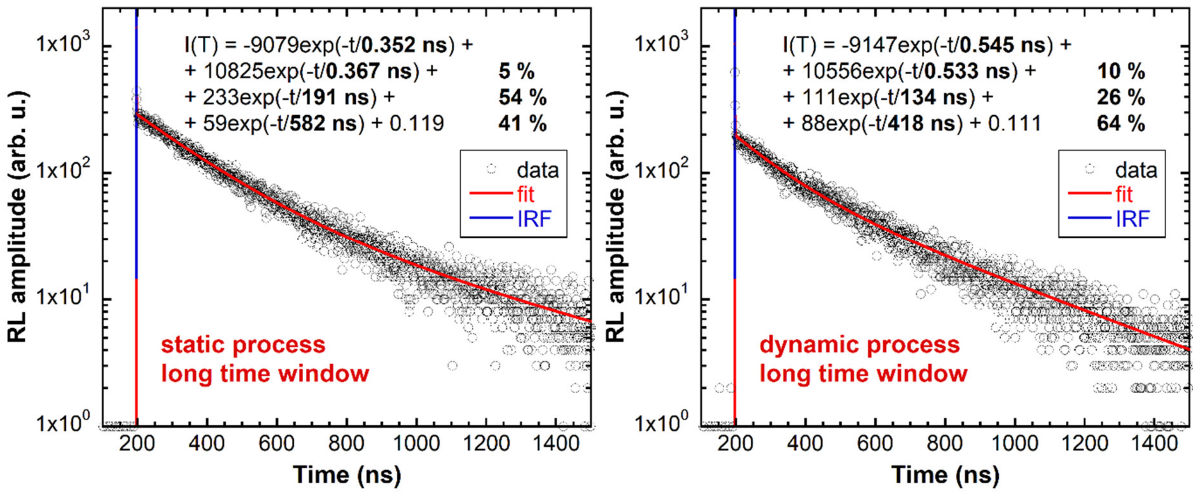

Figure S6. Scintillation decays in the long time window of $CsPbBr_3$ thin films on GGAG:Ce prepared by the static process (left) and the dynamic process (right). Black circles represent experimental data, red line represents the fit and blue line is the instrumental response function (IRF).

Literature

29. Berger, M.J.; Hubbell, J.H.; Seltzer, S.M.; Chang, J.; Coursey, J.S.; Sukumar, R.; Zucker, D.S.; Olsen, K. **2010**, XCOM: Photon Cross Section Database (version 1.5). Available online: http://physics.nist.gov/xcom (accessed on 2021, November 9). National Institute of Standards and Technology, Gaithersburg, MD.
